# Strong Biomimetic Immobilization of Pt-Particle Catalyst on ABS Substrate Using Polydopamine and Its Application for Contact-Lens Cleaning with H_2_O_2_

**DOI:** 10.3390/nano10010114

**Published:** 2020-01-07

**Authors:** Yuji Ohkubo, Tomonori Aoki, Daisuke Kaibara, Satoshi Seino, Osamu Mori, Rie Sasaki, Katsuyoshi Endo, Kazuya Yamamura

**Affiliations:** 1 Graduate School of Engineering, Osaka University, Suita, Osaka 565-0871, Japan; t-aoki@div1.upst.eng.osaka-u.ac.jp (T.A.); d-kaibara@div1.upst.eng.osaka-u.ac.jp (D.K.); seino@mit.eng.osaka-u.ac.jp (S.S.); endo@upst.eng.osaka-u.ac.jp (K.E.); yamamura@prec.eng.osaka-u.ac.jp (K.Y.); 2 Menicon Co., Ltd., Kasugai, Aichi 487-0032, Japan; o-mori@menicon.co.jp (O.M.); r-sasaki@menicon.co.jp (R.S.)

**Keywords:** catalytic durability, polydopamine (PDA), strong adhesion, supported catalyst, H_2_O_2_ decomposition

## Abstract

Polydopamine (PDA)—a known adhesive coating material—was used herein to strongly immobilize a Pt-particle catalyst on an acrylonitrile–butadiene–styrene copolymer (ABS) substrate. Previous studies have shown that the poor adhesion between Pt particles and ABS surfaces is a considerable problem, leading to low catalytic durability for H_2_O_2_ decomposition during contact-lens cleaning. First, the ABS substrate was coated with PDA, and the PDA film was evaluated by X-ray photoelectron spectroscopy. Second, Pt particles were immobilized on the PDA-coated ABS substrate (ABS-PDA) using the electron-beam irradiation reduction method. The Pt particles immobilized on ABS-PDA (Pt/ABS-PDA) were observed using a scanning electron microscope. The Pt-loading weight was measured by inductively coupled plasma atomic emission spectroscopy. Third, the catalytic activity of the Pt/ABS-PDA was evaluated as the residual H_2_O_2_ concentration after immersing it in a 35,000-ppm H_2_O_2_ solution (the target value was less than 100 ppm). The catalytic durability was evaluated as the residual H_2_O_2_ concentration after repeated use. The PDA coating drastically improved both the catalytic activity and durability because of the high Pt-loading weight and strong adhesion among Pt particles, PDA, and the ABS substrate. Plasma treatment prior to PDA coating further improved the catalytic durability.

## 1. Introduction

Mussels can strongly adhere to several surfaces using their body fluid, regardless of whether the surfaces are dry or wet [1,2,3]. This phenomenon of adhesion to wet surfaces is unusual in the adhesives industry. The body fluid of mussels was examined, and it was found that 3,4-dihydroxy-L-phenylalanine (DOPA) and lysine-enriched proteins contributed to its strong adhesion [4,5,6]. As a result, polydopamine (PDA) has attracted the attention of many scientists because its structure is similar to that of DOPA. Surface chemical composition affects adhesion properties. Both DOPA and PDA have hydroxyl and amino groups and a benzene ring, so they can interact with various materials such as metal oxides, metals, and polymers, not only through van der Waals forces, but also via hydrogen or coordinate bonding or π–π stack interaction. Since PDA has been reported as a novel adhesive coating for several materials such as Pt, Cu, TiO_2_, SiO_2_, and Al_2_O_3_ [7], it has received even more attention. For example, there are reports of PDA being utilized at sites for growing hydroxyapatite (HAp) [8]; PDA-coated polystyrene (PS) particles have been used to prepare a structural-color-controlled ink [9]; a PDA-grafted hydrogel has been demonstrated to adhere to a wet mucous membrane [10]; and a PDA coating has been used as a seed layer for a TiO_2_–polytetrafluorethylene (PTFE) nanocomposite coating [11]. Recently, PDA was utilized to improve the adhesion between a polydimethylsiloxane (PDMS) nanosheet and a living body [12]. This research demonstrated that a PDA coating combines both high adhesion and biocompatibility.

**Figure 1:**
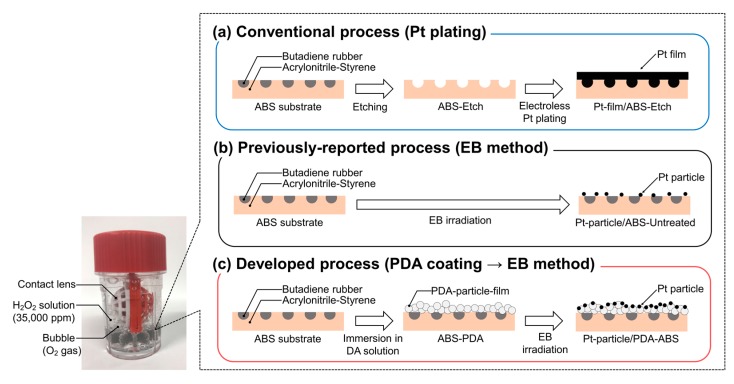
Schematic of the processes for preparing (**a**) a Pt-film/ABS sample by the electroless plating method, (**b**) a Pt-particle/ABS sample by EBIRM without pre-treatment, and (**c**) a Pt-particle/ABS-PDA sample by EBIRM adding a PDA coating as a pre-treatment.

The number of contact-lens wearers has increased in recent years and is currently estimated to be approximately 140 million [13]. There are two types of contact-lens wearers, namely, those who use one-day disposable lenses and those who prefer repeatable-use (e.g., monthly) contact lenses. Although one-day disposable contact lenses do not need cleaning and disinfecting, their high cost is a serious disadvantage. In contrast, repeatable-use contact lenses are less expensive in the long term, but it is essential to clean and disinfect them properly once per day to prevent eye infections. There are three types of methods for cleaning and disinfecting contact lenses: the first method is boil cleaning, the second is H_2_O_2_ cleaning, and the third is cleaning with a multipurpose solution (MPS). MPS cleaning has the advantage of being simple because only one solution is required for cleaning, disinfection, and storage. However, contact-lens wearers applying the MPS cleaning method are likely to have eye problems if they do not clean their contact lenses carefully enough. Thus, the number of contact-lens wearers using MPS cleaning has gradually decreased since 2009, while that of users applying the H_2_O_2_ cleaning method has increased [14]. The reason for this is that eye problems are unlikely to occur in the case of H_2_O_2_ cleaning because a 35,000-ppm H_2_O_2_ solution exhibits a high disinfecting performance. However, when H_2_O_2_ cleaning is applied, there is a risk of the eyes becoming bloodshot or painful—even of blindness—if the 35,000-ppm H_2_O_2_ solution enters the eyes without being decomposed to a concentration of 100 ppm [15]. Electroless platinum (Pt) plating has been performed to give an acrylonitrile–butadiene–styrene copolymer (ABS) substrate catalytic performance for accelerating the H_2_O_2_ decomposition process (Figure 1a). Pt is very expensive, so there is a strong need to decrease the amount of Pt used in contact-lens cleaners. We have suggested replacing the Pt film with Pt particles, which results in a drastic decrease in the amount of Pt required (Figure 1b) [16]. However, some problems remain regarding the catalytic durability, although we pretreated the surface by etching, electric charge control, or both [17]. As mentioned above, PDA has the potential to adhere metal particles to resin substrates. In this study, we used a PDA coating as a pre-treatment to strongly and safely immobilize Pt particles on an ABS substrate to improve the catalytic durability of the material (Figure 1c). The effects of the PDA coating on the properties of the ABS surface and the catalytic activity, Pt-loading weight, and catalytic durability of the system were investigated.

## 2. Results and Discussion

### 2.1. External Appearance

**Figure 2:**
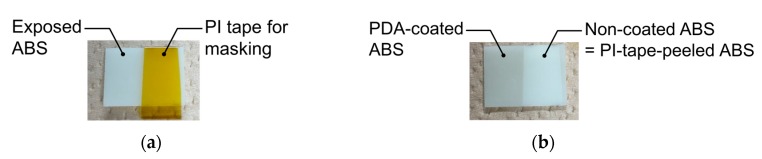
Photographs of ABS samples with a masking using polyimide (PI) tape (**a**) before PDA coating and (**b**) after PDA coating for 24 h.

The changes in the external appearances of the ABS samples were monitored to confirm that PDA coating had occurred. Figure 2 presents photographs of ABS samples with a masking using polyimide (PI) tape before and after PDA coating. The color of the PDA-coated area changed from cream to gray, and this gray color remained after ultrasonic cleaning, which confirmed that the PDA film was strongly attached to the ABS surface.

### 2.2. Confirmation of the Formation of a PDA Film by X-Ray Photoelectron Spectroscopy (XPS)

**Figure 3:**
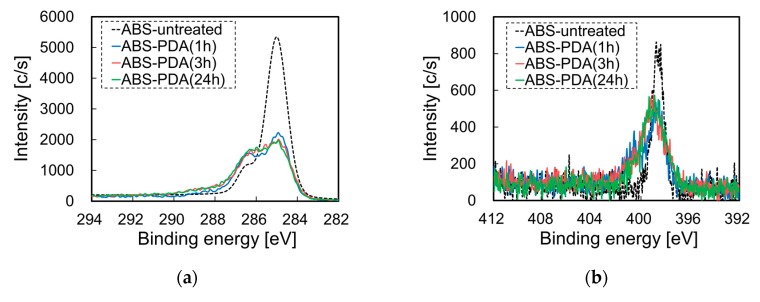

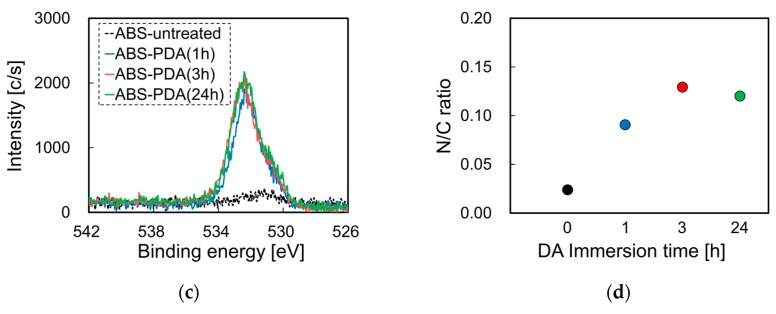
X-ray photoelectron spectroscopy (XPS) spectra of an ABS surface before and after PDA coating at different immersion times in a DA solution: (**a**) C1s-XPS, (**b**) N1s-XPS, (**c**) O1s-XPS, and (**d**) the N/C ratio.

To examine the effects of the PDA coating on the chemical composition of ABS substrates, we analyzed pretreated ABS surfaces that did not contain Pt particles by XPS. Figure 3 presents the XPS spectra of the surface of an ABS substrate before and after PDA coating at different immersion times in a dopamine (DA) solution. When the ABS substrates were immersed in a DA solution, the intensities of the peaks assigned to C–H and C–C (285 eV) decreased, whereas those of the peaks assigned to C–N and C–O (286.5 eV) increased, as illustrated in Figure 3a. The intensities of the signals in the N1s-XPS spectra did not increase because the ABS substrate originally contained a C≡N bond, as illustrated in Figure 3b. In addition, when the ABS substrates were immersed in a DA solution, the intensities of the signals in the O1s-XPS spectra also increased, as illustrated in Figure 3c. The calculated N/C atomic ratio is presented in Figure 3d, where it can be seen that it increased with increasing immersion time in the DA solution. When the ABS substrates were immersed in a DA solution for 3 and 24 h, the N/C ratios were 0.129 and 0.120, respectively. These ratios were roughly consistent with the theoretical value of N/C = 0.125. These results indicate that the ABS surfaces were coated with a PDA film.

### 2.3. Observation of Pt Particles by SEM

**Figure 4:**
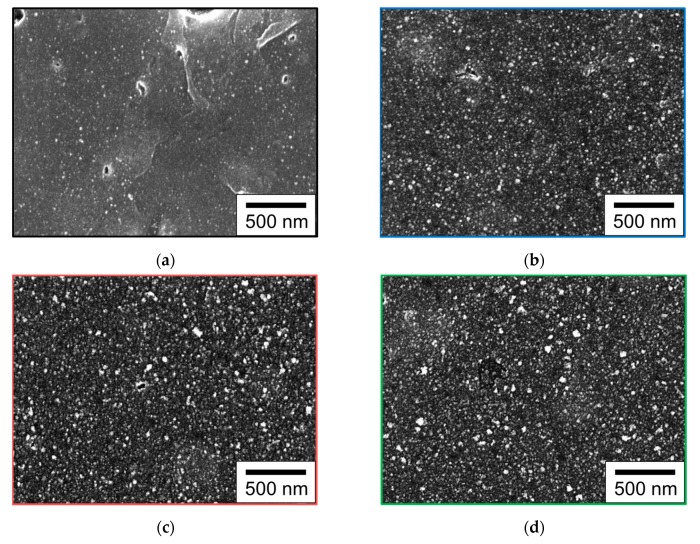
Scanning electron microscope (SEM) images of the surface of an ABS substrate before and after PDA coating and Pt immobilization: (**a**) Pt/ABS-untreated, (**b**) Pt/ABS-PDA(1h), (**c**) Pt/ABS-PDA(3h), and (**d**) Pt/ABS-PDA(24h).

The Pt particles immobilized on the ABS surfaces were analyzed by SEM to confirm the deposition of Pt particles. Figure 4 presents SEM micrographs of the surfaces of Pt/ABS samples with or without PDA coating at a low magnification. The small white spots in the images are Pt particles. The high dispersibility of Pt particles was confirmed for all samples. The number of Pt particles clearly increased upon PDA coating. Moreover, the number of Pt particles increased with increasing DA immersion time. Some holes (with diameters of 50–200 nm) were observed in the Pt/ABS-untreated sample (Figure 4a), but they were absent in the Pt/ABS-PDA samples (Figure 4b–d). This indicates that the holes originally present on the ABS surface were successfully coated with the PDA film.

### 2.4. Pt-Loading Weight Determined by Inductively Coupled Plasma Atomic Emission Spectrometry (ICP-AES)

**Figure 5:**
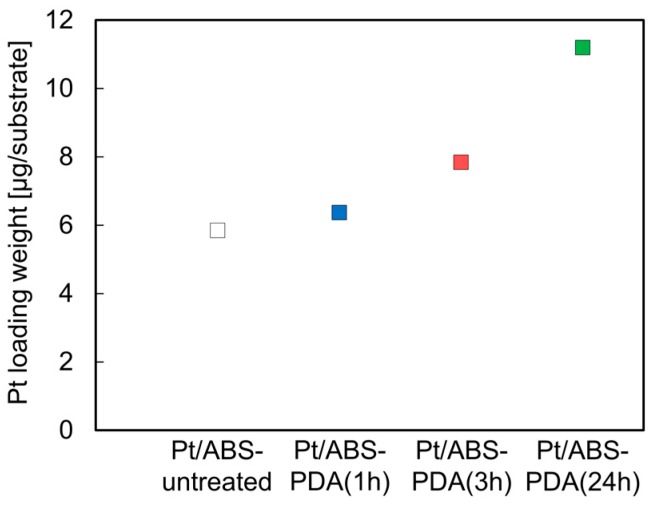
Pt-loading weights of Pt/ABS samples with or without the PDA coating.

The effects of the PDA coating on the Pt-loading weights of the Pt/ABS samples were also examined. Figure 5 illustrates the Pt-loading weights of substrates with or without PDA coating. It can be seen that the values were higher for the Pt/ABS-PDA samples than for the Pt/ABS-untreated ones, thus indicating that PDA coating increased the Pt-loading weight. In addition, the Pt-loading weights for the Pt/ABS-PDA samples increased with increasing DA immersion time. These results of Pt-loading weight are consistent with the SEM images illustrated in Figure 4. When an ABS substrate is coated with a PDA film, the number of sites for immobilizing Pt particles also increases, resulting in an increased Pt-loading weight. The Pt-loading weight of the Pt/ABS-PDA(24h) material (11.2 μg/substrate) was approximately twice that of the Pt/ABS-untreated sample, but at least 130 times lower than that of an ABS substrate coated with an electroless-plated Pt film (Pt-film/ABS) (1500 μg/substrate), which had been studied earlier [16].

### 2.5. Catalytic Activity for H_2_O_2_ Decomposition

**Figure 6:**
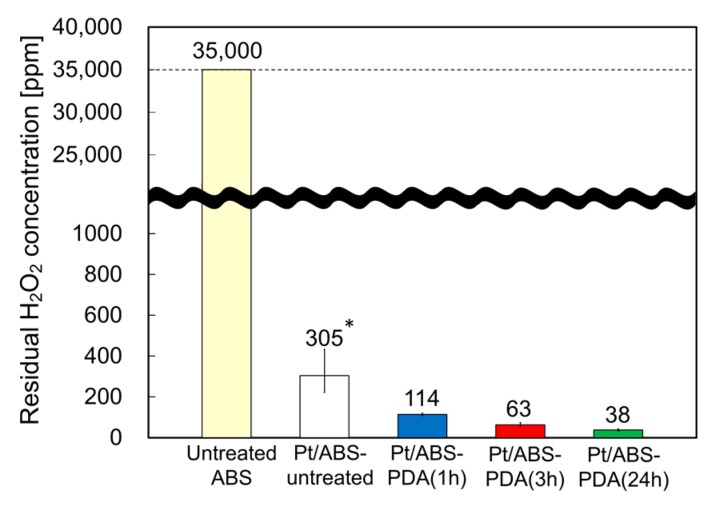
Catalytic activity of Pt/ABS samples with or without PDA coating: residual H_2_O_2_ concentration after immersion for 360 min. * The data for the Pt/ABS-untreated are the same as in our previous report [17].

To evaluate the catalytic activity of the materials for H_2_O_2_ decomposition, the residual H_2_O_2_ concentration was measured in the system after immersing Pt/ABS samples with or without the PDA coating in a 35,000-ppm H_2_O_2_ solution for 360 min. Briefly, the lower the residual H_2_O_2_ concentration, the higher the catalytic activity. Figure 6 illustrates the catalytic activity of Pt/ABS samples with or without the PDA coating. The untreated ABS sample without Pt particles did not decompose H_2_O_2_ within 360 min, whereas all the samples with Pt immobilized on the ABS substrate significantly decreased the residual H_2_O_2_ concentration from 35,000 to less than 400 ppm. Moreover, the residual H_2_O_2_ concentrations for the Pt/ABS-PDA samples became lower than that for the Pt/ABS-untreated sample. It is clear that the PDA coating improved the catalytic activity for H_2_O_2_ decomposition, as well as that the residual H_2_O_2_ concentration decreased with increasing DA immersion time. This result is consistent with those obtained in the SEM (Figure 4) and ICP-AES (Figure 5) studies. In summary, an increase in the Pt-loading weight contributed to the improvement of the catalytic activity of the resulting material. The target value for the residual H_2_O_2_ concentration (i.e., <100 ppm) was successfully reached after 3 h of immersion in DA.

### 2.6. Catalytic Durability during H_2_O_2_ Decomposition

**Figure 7:**
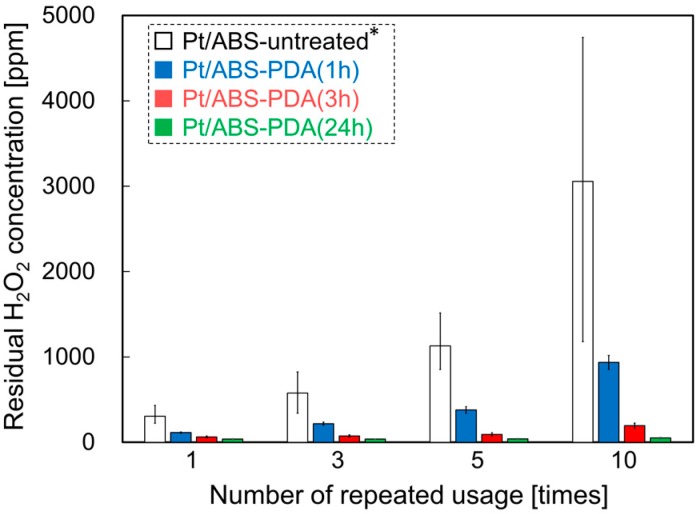
Catalytic durability of Pt/ABS samples with or without PDA coating: the relationship between the number of usage cycles and the residual H_2_O_2_ concentration. * The data for the Pt/ABS-untreated are the same as in our previous report [17].

To examine the effect of the PDA coating on the catalytic durability of the system, the relation between the number of repeated uses and the residual H_2_O_2_ concentration was examined. Figure 7 illustrates the catalytic durability of Pt/ABS samples with or without the PDA coating. The residual H_2_O_2_ concentration for the Pt/ABS-untreated sample increased significantly with increasing usage, thus resulting in low catalytic durability. In the case of the Pt/ABS-PDA material, the residual H_2_O_2_ concentrations measured after using the samples 10 times were 935, 194, and 54 ppm for Pt/ABS-PDA(1h), Pt/ABS-PDA(3h), and Pt/ABS-PDA(24h), respectively; this indicates that the residual H_2_O_2_ concentrations decreased with increasing DA immersion time. This result demonstrates that the PDA coating effectively improved the catalytic durability of the material for H_2_O_2_ decomposition. Moreover, the residual H_2_O_2_ concentration for Pt/ABS-PDA(24h) was still below 100 ppm after the catalyst had been used 10 times.

### 2.7. Effect of Plasma Treatment on Catalytic Durability

**Figure 8:**
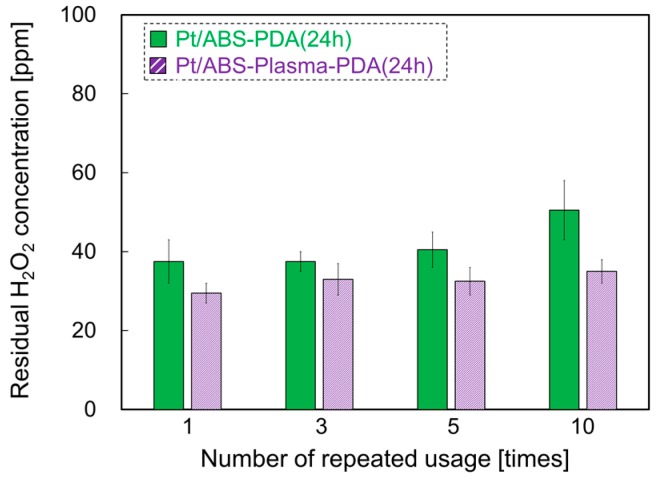
Catalytic durability of Pt/ABS-PDA(24h) samples with or without plasma treatment before PDA coating: the relationship between the number of usage cycles and the residual H_2_O_2_ concentration.

Although Pt/ABS-PDA(24h) exhibited high catalytic durability, the residual H_2_O_2_ concentration mildly increased from 38 to 51 ppm with the number of usage cycles. Thus, to further improve the catalytic durability, plasma treatment was applied before the PDA coating. Figure 8 illustrates the catalytic durability of Pt/ABS-PDA(24h) samples with or without plasma treatment. Please note that the range of the vertical axis is from 0 to 100 ppm. The residual H_2_O_2_ concentration of the Pt/ABS-plasma-PDA(24h) sample after it had been used 10 times was 35 ppm, which indicates that the residual H_2_O_2_ concentration barely changed and that the plasma treatment before the PDA coating further improved the catalytic durability of the material. The catalytic durability of the Pt/ABS-plasma-PDA(24h) sample was sufficient for use in practical applications.

**Figure 9:**
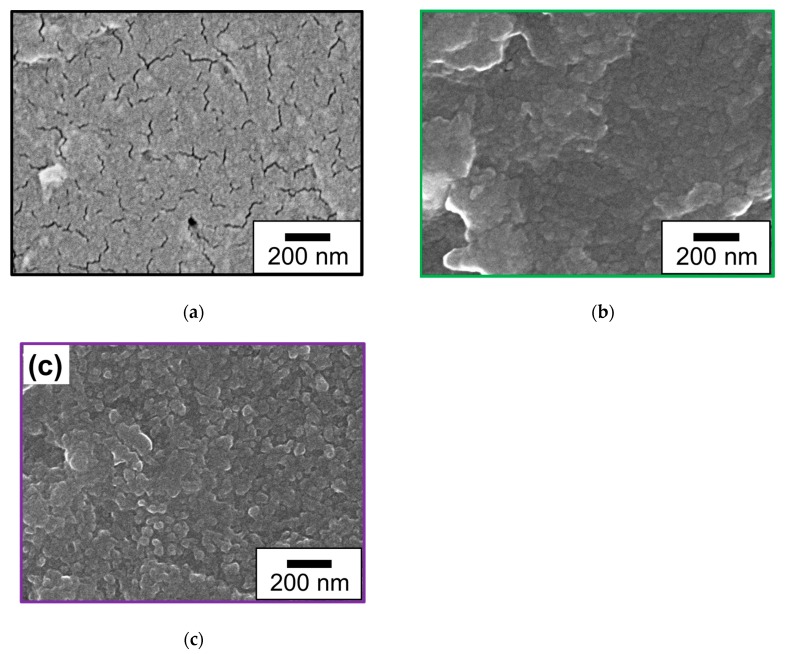
Scanning electron microscope (SEM) images of the surface of an ABS substrate before and after PDA coating with or without plasma treatment: (**a**) ABS-untreated, (**b**) ABS-PDA(24h), and (**c**) Pt/ABS-plasma-PDA(24h).

**Figure 10:**
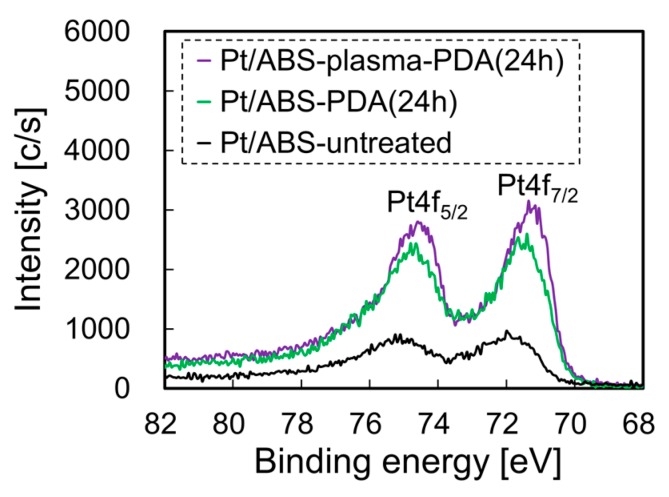
Pt4f-XPS spectra of the Pt/ABS samples with or without plasma treatment and with or without PDA coating.

To investigate the effect of the plasma treatment on the morphology of the PDA coating, the surfaces of the ABS-PDA(24h) and ABS-plasma-PDA(24h) samples were observed and compared by SEM. Figure 9 presents SEM images of the surface of an ABS substrate before and after PDA coating, with or without plasma treatment. Although many cracks were observed on the as-received ABS surface, no cracks were observed on the PDA-coated one, regardless of the plasma treatment. This result indicates that the ABS surfaces were coated with a PDA film. A comparison between the ABS-plasma-PDA(24h) and ABS-PDA(24h) samples indicates that the surface roughness of the plasma-treated material was larger than that of the untreated one. The Pt-loading weights of Pt/ABS-plasma-PDA(24h) and Pt/ABS-PDA(24h) were 24.0 and 11.2 µg, respectively. As can be seen, the plasma treatment induced an increase in the Pt-loading weight, which is one of the reasons for the improved catalytic activity and durability. In addition, XPS measurements were also carried out for the ABS samples after Pt immobilization to investigate the effect of plasma treatment on the Pt state, such as Pt(0), Pt(II), and Pt(IV). Figure 10 presents the Pt4f-XPS spectra of the Pt/ABS samples with or without plasma treatment and with or without PDA coating. Two broad peaks indexed to Pt4f_7/2_ and Pt4f_5/2_ were observed for all the Pt4f-XPS spectra. The Pt4f_7/2_ peak was resolved into three peaks indexed to Pt(0), Pt(II), and Pt(IV) at ca. 71.6, ca. 72.6, and 74.4 eV, respectively [18,19]. The ratios of Pt(0), Pt(II), and Pt(IV) are listed in Table 1 according to the references. Although the main state was Pt(0), minor states of Pt(II) and Pt(IV) also were detected. These results indicate two likelihoods: the first is a coordinate bond of Pt ions, and the second is an oxidation of Pt. In summary, it is possible that, firstly, Pt in an ionic state interacted with C–O groups on the electron-beam-irradiated ABS substrate in water or NH_2_ and N–H groups in the PDA film; secondly, a coordinate bond was formed; thirdly, Pt clusters grew to form Pt particles; and finally, the surface of the Pt particles were oxidized to change the surface to Pt oxide (PtO or PtO_2_). The PDA coating increased the ratio of the Pt metallic state to >80%, while plasma treatment mostly did not affect the ratio of the Pt metallic state.

## 3. Materials and Methods

### 3.1. PDA Coating and Immobilization of Pt Particles on ABS

Pt particles were immobilized on an ABS substrate by the electron-beam-irradiation reduction method (EBIRM) because of the advantages of this procedure, which include a low processing temperature, highly uniform deposition, and a high throughput [20]. Although there are many methods for preparing and immobilizing particles (e.g., sonolytic [21,22,23,24], polyol [25,26,27], and impregnation [28,29,30] methods), they all have disadvantages such as high processing temperatures, nonuniform deposition of the metal particles, and low throughput. Therefore, EBIRM was selected in this study. The mechanism of metal ion reduction by a radiochemical approach has been described in previous reports [31,32], and the methods applied for washing the ABS substrate and depositing the Pt particles on its surface were the same here as those reported previously [16,17]. The differences between this report and previous ones are the plasma pre-treatment and the use of a PDA coating to improve the catalytic durability during H_2_O_2_ decomposition.

A commercially available ABS sheet (thickness *t* = 1 mm, 2-9229-01, AS-ONE, Nishi-ku, Osaka, Japan) was cut into substrates with a size of 20 × 15 mm^2^. The ABS substrates were first washed with ethanol (99.5%, Kishida Chemical, Chuo-ku, Osaka, Japan) and pure water for 10 min each in an ultrasonic cleaner (USK-1R, AS-ONE) and then dried through blowing N_2_ gas (99.99%, Iwatani Fine Gas, Amagasaki, Hyogo, Japan). Prior to immobilizing the Pt particles, the washed substrates were pretreated either by only PDA coating or both plasma treatment and PDA coating. Table 2 presents the sample conditions and IDs.

Low-pressure plasma treatment was applied at 100 Pa using a plasma chamber (PR-501A, Yamato Scientific, Chuo-ku, Tokyo, Japan) with a radio frequency power source of 13.56 MHz. Before the plasma treatment, the washed ABS substrates were placed in the plasma chamber; subsequently, the pressure in the chamber was decreased to 5 Pa using a rotary vacuum pump (2012AC, Alcatel Vacuum Technology, Annecy, France). Then, helium gas (99.99%, Iwatani Fine Gas) flowed into the chamber until its pressure reached 100 Pa. The applied power for plasma generation was 100 W, and the standing wave ratio was controlled at less than 1.1 through impedance matching. The plasma treatment time was 60 s. XPS measurements were performed to confirm that the surface of the ABS substrate was modified by the plasma treatment. The results confirmed that oxygen-containing functional groups (–O–C=O and –C–O) were generated by the plasma treatment, as illustrated in Figure S1.

Aqueous solutions (20 mL) containing 2 mg/mL of DA were prepared using 2-(3,4-dihydroxyphenyl) ethylamine hydrochloride (C_8_H_12_NO_2_·HCl; 98%, Fujifilm Wako Pure Chemical, Chuo-ku, Osaka, Japan) as the dopamine precursor and Tris hydrochloride acid buffer, controlled at pH = 8.5 (1 mol/L, Fujifilm Wako Pure Chemical), as the solvent. The ABS substrates were immersed in the DA solution for different times (i.e., 1, 3, or 24 h), and the DA solutions were neither stirred nor bubbled with oxygen gas during the immersion. After PDA coating, the substrates were removed from the DA solution and washed with pure water for 10 min using an ultrasonic cleaner to remove the unreacted DA and PDA. Finally, they were dried with N_2_ gas. Precleaned slide glasses (S7213, Matsunami Glass, Kishiwada, Osaka, Japan) were also used and coated with a PDA film to confirm that the PDA coating was completed because ABS substrates originally contain carbon and nitrogen atoms. The results demonstrated that the intensity of the peaks in the Si2p-XPS spectra decreased with increasing DA immersion time and eventually disappeared when the glass slide was immersed for 24 h, as illustrated in Figure S2. This result indicates that immersion in a DA solution for 24 h is enough to uniformly cover the substrate’s surface.

Aqueous solutions (5 mL) containing 4 mM of Pt ions were prepared using hexachloroplatinic acid hexahydrate (H_2_PtCl_6_·6H_2_O; 98.5%, Fujifilm Wako Pure Chemical) in cylindrical PS containers (diameter ⌀ = 33 mm and height *h* = 16 mm). Then, 2-propanol (IPA; 99.7%, Kishida Chemical) was added to the Pt ion solution (to be controlled at 1 vol %), and the pretreated ABS substrate was immersed in the Pt precursor solutions. A high-energy electron beam (of 4.8 MeV) was irradiated on these Pt precursor solutions containing the pretreated ABS substrate for 7 s using a Dynamitron^®^ accelerator from SHI-ATEX Co. Ltd. (Izumiotsu, Osaka, Japan). During electron-beam irradiation, H radicals and hydrated electrons were generated by the radiolysis of water. The reductive species reduced Pt^4+^ ions to Pt^3+^, Pt^2+^, Pt^1+^, and Pt^0^, as described in a previous report [31]. Subsequently, clusters of Pt atoms were formed, which grew to produce Pt particles. The Pt particles were formed not only on the substrate, but also in the solution. When the Pt particles grew from Pt clusters on the substrate, they were immobilized on it. When the Pt particles grew from Pt clusters in the solution, they fell down and were deposited on the substrate, but were not immobilized on it. To remove the unimmobilized Pt particles, the ABS substrates were taken out of the solution, washed with pure water for 10 min using an ultrasonic cleaner, and finally dried with N_2_ gas. In a previous report [16], XPS and water contact angle (WCA) measurements were performed for untreated ABS substrates before and after electron beam irradiation to investigate the immobilization mechanism; however, this mechanism is not yet clear. Those XPS and WCA results suggest two models: the first model assumes the chemical adhesion of Pt nanoparticles through a chemical reaction of functional groups (C–O) and/or carbon radicals with Pt ions and/or Pt^0^, and the second model assumes the unreactive immobilization of Pt nanoparticles through a C–C crosslinking network under EB irradiation. To comprehensively clarify the immobilization mechanism, further experiments using a simplex polymer such as polyethylene should be conducted.

### 3.2. Characterization

To confirm that the ABS surface was coated with a PDA film, its chemical composition was determined by XPS using Quantum 2000 equipment (Ulvac-Phi, Chigasaki, Kanagawa, Japan) attached to an Al-*K*α source at 15 kV. The diameter of X-ray irradiation was ⌀ = 100 μm; the pass energy and step size were 23.50 and 0.05 eV, respectively; and the take-off angle was 45°. To neutralize the electric charges on the surfaces, the measured samples were irradiated with a low-speed electron beam and an Ar ion beam during the XPS measurements.

Secondary electron images using a field-emission scanning electron microscope (FE-SEM; S-4800, Hitachi High-Technologies Corporation, Minato-ku, Tokyo, Japan) at 5 kV of accelerated voltage were obtained to monitor the deposition behavior of the Pt particles on the ABS and/or ABS-PDA surfaces. Prior to the observations, osmium (Os) was coated on the Pt/ABS surfaces by plasma chemical vapor deposition using an osmium plasma coater (OPC60AL, Filgen, Nagoya, Aichi, Japan) to prevent the generation of electrostatic charges during the measurements. The same FE-SEM instrument was also used to investigate the effect of the plasma treatment on the film-forming state of PDA.

To measure the Pt-loading weight, the Pt particles on the ABS and/or PDA surfaces were dissolved in aqua regia, which was prepared by mixing hydrochloric acid (HCl; 35%, Sigma-Aldrich Japan, Meguro-ku, Tokyo, Japan) and nitric acid (HNO_3_; 69%, Sigma-Aldrich Japan) at a ratio of 3:1. Then, the Pt concentrations were measured by ICP-AES (ICPE-9000, Shimadzu, Chukyo-ku, Kyoto, Japan) using the diluted aqua regia solutions containing Pt ions. A calibration curve prepared with a standard Pt solution (1000 ppm, Fujifilm Wako Pure Chemical) was used to calculate the amount of Pt in the Pt/ABS samples, as illustrated in Figure S3.

**Figure 11:**
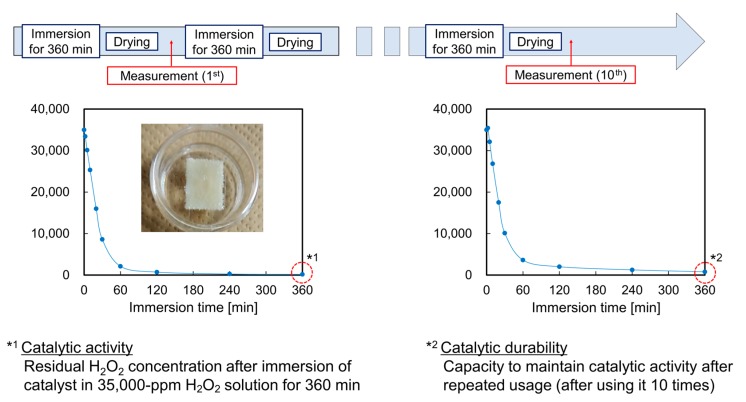
Schematic of the process for evaluating the catalytic activity and durability of the system using typical H_2_O_2_ decomposition curves. *^1^ Value of the residual H_2_O_2_ concentration for evaluating the catalytic activity. *^2^ Value of the residual H_2_O_2_ concentration for evaluating the catalytic durability.

Figure 11 is a schematic of the process for evaluating the catalytic activity and durability of the system using representative H_2_O_2_ decomposition curves. First, the Pt/ABS samples were immersed in 5 mL of a 35,000-ppm solution of H_2_O_2_ (30 wt%, Kishida Chemical) at 25 °C for 360 min in an incubator (i-CUBE FCI-280, AS-ONE, Nishi-ku, Osaka, Japan). H_2_O_2_ decomposition occurred with increasing immersion time. The residual H_2_O_2_ concentrations were measured after immersion times of 1, 2, 5, 10, 30, 60, 120, 240, and 360 min to obtain the decomposition curves. The method for measuring the H_2_O_2_ concentration was the same as that reported in a previous article [16]. The optical absorbance of a H_2_O_2_ solution, colored using diluted (5 wt%) titanium sulfate (Ti(SO_4_)_2_; 30 wt%, Fujifilm Wako Pure Chemical), was measured using a deuterium–halogen and tungsten lamp (DH-2000, Ocean Optics, Largo, FL, USA), a fiber multichannel spectrometer (HR-4000, Ocean Optics), and optical fiber (P600-1-UV/VIS, Ocean Optics). The absorbance at 407 nm was used to calculate the residual H_2_O_2_ concentration from the calibration curve, as presented in Figure S4. The catalytic activity for H_2_O_2_ decomposition was evaluated from the value of the residual H_2_O_2_ concentration after immersing the Pt/ABS samples in the H_2_O_2_ solution for 360 min. This process of immersing the catalyst in the H_2_O_2_ solution for 360 min and drying it was repeated 10 times. The residual H_2_O_2_ concentrations were measured after one, three, five, and 10 immersions. The catalytic durability for H_2_O_2_ decomposition was evaluated from the value of the residual H_2_O_2_ concentration after immersing the Pt/ABS samples in the H_2_O_2_ solution 10 times (for 360 min each). The target value was less than 100 ppm, which means that if the residual H_2_O_2_ concentration was below 100 ppm after repeated use (i.e., after 10 uses), the Pt/ABS sample had long catalytic durability.

## 4. Conclusions

We introduced PDA coating as a pre-treatment for the strong immobilization of Pt-particle catalysts on ABS substrates and investigated the effect of the PDA coating on the deposition behavior of the Pt particles, the Pt-loading weight, and the catalytic activity and durability of the material. We found that the PDA coating improved both the catalytic activity and durability of the Pt-based material. Moreover, introducing a plasma treatment before the PDA coating was effective for further improving the catalytic durability. Finally, in the case of the Pt/ABS-plasma-PDA(24h) catalyst, the residual H_2_O_2_ concentrations were 30, 33, 33, and 35 ppm after using the material 1, 3, 5, and 10 times, respectively. Although the PDA coating also increased the Pt-loading weight (from 5.9 to 24.0 μg/substrate), the value measured for the Pt-particle/ABS-plasma-PDA(24h) catalyst (i.e., 24.0 μg/substrate) was significantly below that determined for a Pt-film/ABS catalyst (1500 μg/substrate) prepared by electroless plating. In summary, we successfully achieved a decrease in Pt usage while maintaining the high catalytic activity and durability. In addition, the developed process, which includes a combination of plasma treatment, PDA coating, and EBIRM, does not require etching of the ABS surface using dangerous chemical solutions, as is the case for electroless plating, where previous etching is necessary to obtain high adhesion between the ABS substrate and the Pt film. Therefore, the developed process is more ecofriendly. Although the PDA coating was used to improve the catalytic durability of a Pt-based catalyst in this study, this type of coating is useful as a pre-treatment for the strong immobilization of metal particles on several substrates or microparticles.

## Figures and Tables

**Table 1:** Ratios of Pt(0), Pt(II), and Pt(IV) calculated from peak resolution of the Pt4f_7/2_ in Figure 10.

Sample ID	Pt(0)	Pt(II)	Pt(IV)
Pt/ABS-untreated	69.1	16.4	14.5
Pt/ABS-PDA(24h)	81.1	11.0	7.9
Pt/ABS-plasma-PDA(24h)	86.7	7.1	6.2

**Table 2:** Sample conditions and IDs.

Sample ID	Plasma pre-treatment	PDA coating	Pt-particle deposition
Glass-untreated	―	―	―
Glass-PDA(1h)	―	◯	―
Glass-PDA(3h)	―	◯	―
Glass-PDA(24h)	―	◯	―
ABS-untreated	―	―	―
ABS-PDA(1h)	―	◯	―
ABS-PDA(3h)	―	◯	―
ABS-PDA(24h)	―	◯	―
ABS-plasma	◯	―	―
ABS-plasma-PDA(24h)	◯	◯	―
Pt/ABS-untreated	―	―	◯
Pt/ABS-PDA(1h)	―	◯	◯
Pt/ABS-PDA(3h)	―	◯	◯
Pt/ABS-PDA(24h)	―	◯	◯
Pt/ABS-plasma-PDA(24h)	◯	◯	◯

“―” indicates no operation and “◯” indicates operation.

## Supplementary Materials

The following are available online at https://www.mdpi.com/2079-4991/10/1/114/s1. Figure S1: XPS spectra of the ABS surface before and after plasma treatment, Figure S2: Results of XPS analysis of the glass surface before and after PDA coating, Figure S3: Calibration curve for calculating the amount of Pt on Pt/ABS samples, and Figure S4: Calibration curve for calculating the H_2_O_2_ concentration.
