# Supplementary material for: Strong Biomimetic Immobilization of Pt-Particle Catalyst on ABS Substrate Using Polydopamine and Its Application for Contact-Lens Cleaning with H2O2
Source: Nanomaterials (Basel). 2020 Jan 7;10(1):114. doi: 10.3390/nano10010114 (PMC7022822; doi:10.3390/nano10010114)
Supplement: Supplementary file 1 [file nanomaterials-10-00114-s001.pdf]

Supplementary Materials

Strong biomimetic immobilization of Pt-particle catalyst on ABS substrate using polydopamine and its application for contact-lens cleaning with H_2O_2

Yuji Ohkubo [1,*], Tomonori Aoki [1], Daisuke Kaibara [1], Satoshi Seino [1], Osamu Mori [2], Rie Sasaki [2], Katsuyoshi Endo [1] and Kazuya Yamamura [1]

[1] Graduate School of Engineering, Osaka University, 2-1 Yamadaoka, Suita, Osaka 565-0871, Japan [Tel and Fax: +81-6-6879-7294, and E-mail: okubo@upst.eng.osaka-u.ac.jp]

[2] Menicon Co., Ltd, 5-1-10 Takamoridai, Kasugai, Aichi 487-0032, Japan

Contents

- XPS spectra of the ABS surface before and after plasma treatment ············ Figure S1
- Result of XPS analysis of the glass surface before and after PDA coating ···· Figure S2
- Calibration curve for calculating the amounts of Pt on Pt/ABS samples ······· Figure S3
- Calibration curve for calculating the H_2O_2 concentration ·························· Figure S4

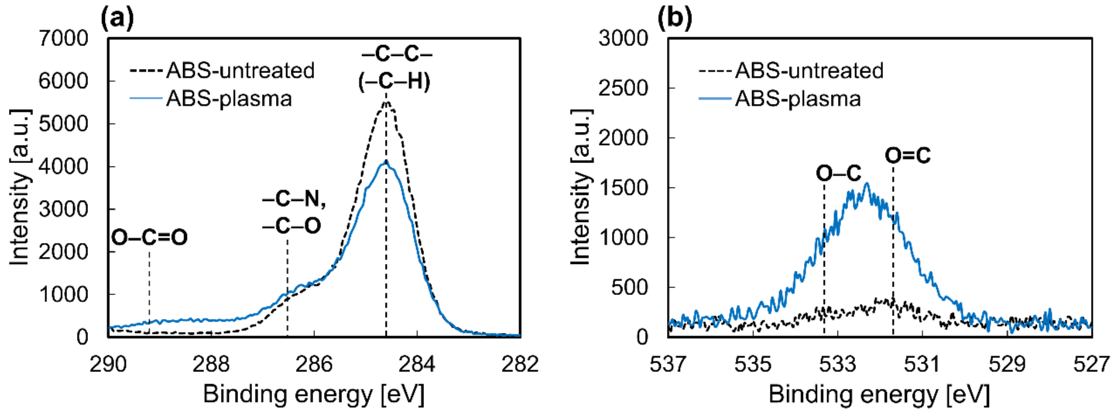

Figure S1. X-ray photoelectron spectroscopy (XPS) spectra of the acrylonitrile–butadiene–styrene copolymer (ABS) surface before and after the plasma treatment: (a) C1s-XPS and (b) O1s-XPS. A helium plasma treatment was applied to the ABS substrate at 100 W and 100 Pa for 60 s. In the case of the C1s-XPS spectra, the intensities of the peaks assigned to –O–C=O (at 289.2 eV) and –C–O (at 286.5 eV) increased as a result of the plasma treatment. In the case of the O1s-XPS spectra, the intensities of the peaks assigned to O–C (at 533.3 eV) and O=C (at 531.7 eV) also increased as a result of the plasma treatment. Thus, it was confirmed that oxygen-containing functional groups were generated during the process.

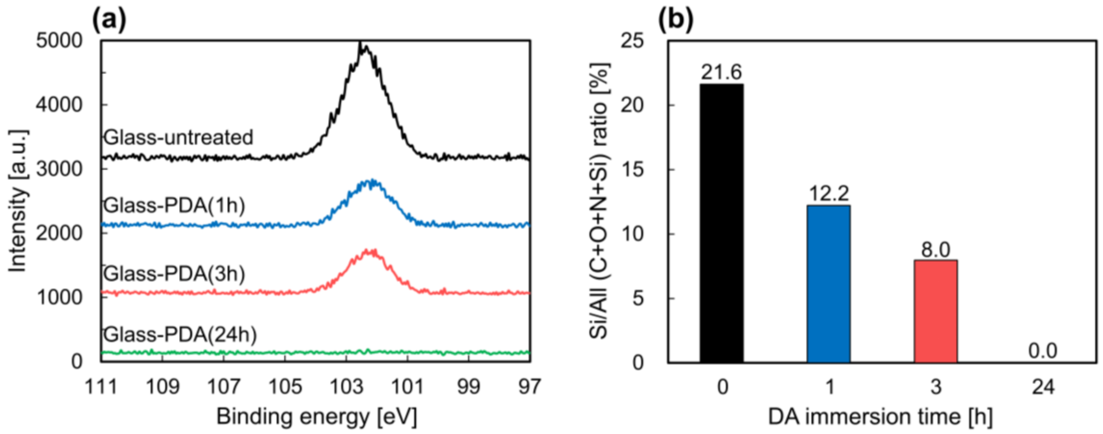

Figure S2. Results of X-ray photoelectron spectroscopy (XPS) analysis of the glass surface before and after PDA coating at different immersion times in a dopamine (DA) solution: (**a**) Si2p-XPS spectra and (**b**) Si/All (C+O+N+Si) ratio. The Si ratio decreased with increasing immersion time in the DA solution. Finally, the Si ratio became 0% when the glass substrate was immersed in the DA solution for 24 h. This result indicates that the glass substrate was uniformly coated with a PDA film (>5 nm in thickness) when it was immersed in the DA solution for 24 h.

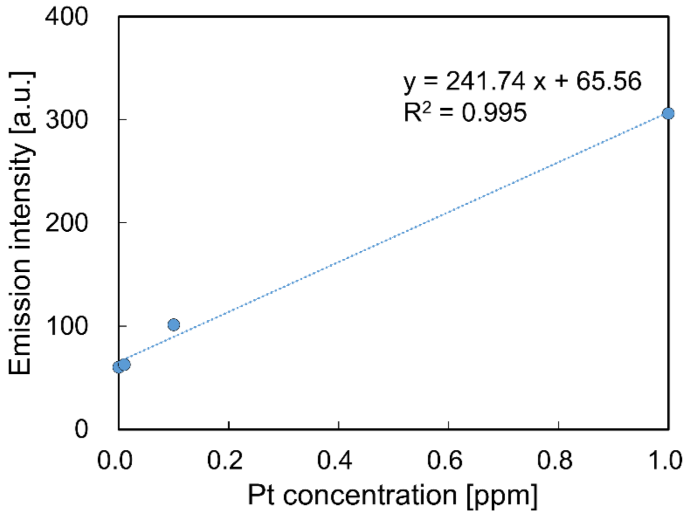

Figure S3. Calibration curve for calculating the amounts of Pt on the Pt/ABS samples: relation between Pt concentration and emission intensity.

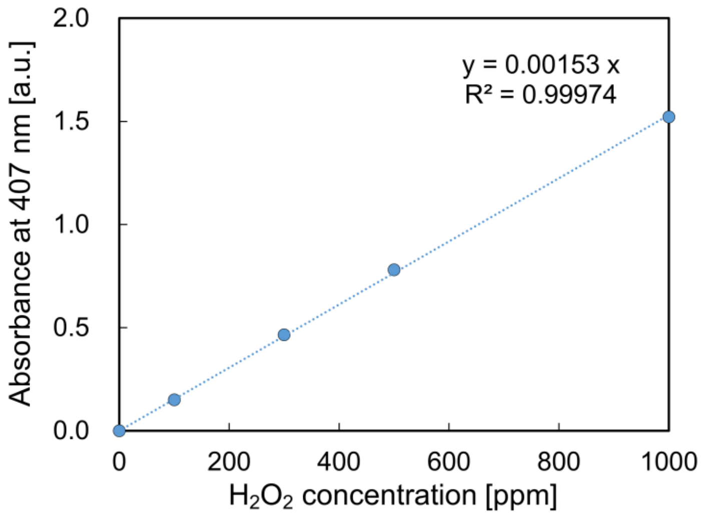

Figure S4. Calibration curve for calculating the H_2O_2 concentration: relation between H_2O_2 concentration and absorbance at 407 nm.
